# Applying Simheuristics to Minimize Overall Costs of an MRP Planned Production System

**DOI:** 10.3390/a15020040

**Published:** 2022-01-27

**Authors:** Wolfgang Seiringer, Juliana Castaneda, Klaus Altendorfer, Javier Panadero, Angel A. Juan

**Affiliations:** 1 School of Business and Management, University of Applied Sciences Upper Austria, Wehrgrabengasse 1-3, 4400 Steyr, Austria; klaus.altendorfer@fh-steyr.at; 2 IN3—Computer Science Department, Universitat Oberta de Catalunya, Rambla Poblenou 156, 08018 Barcelona, Spain; jcastanedaji@uoc.edu (J.C.); jpanaderom@uoc.edu (J.P.); 3 Department of Statistics and OR, Universitat Politècnica de València, Plaza Ferrandiz y Carbonell, 03801 Alcoy, Spain; ajuanp@gmail.com

**Keywords:** MRP, planning parameter, optimization, simulation budget, heuristic

## Abstract

Looking at current enterprise resource planning systems shows that material requirements planning (MRP) is one of the main production planning approaches implemented there. The MRP planning parameters lot size, safety stock, and planned lead time, have to be identified for each MRP planned material. With increasing production system complexity, more planning parameters have to be defined. Simulation-based optimization is known as a valuable tool for optimizing these MRP planning parameters for the underlying production system. In this article, a fast and easy-to-apply simheuristic was developed with the objective to minimize overall costs. The simheuristic sets the planning parameters lot size, safety stock, and planned lead time for the simulated stochastic production systems. The developed simheuristic applies aspects of simulation annealing (SA) for an efficient metaheuristic-based solution parameter sampling. Additionally, an intelligent simulation budget management (SBM) concept is introduced, which skips replications of not promising iterations. A comprehensive simulation study for a multi-item and multi-staged production system structure is conducted to evaluate its performance. Different simheuristic combinations and parameters are tested, with the result that the combination of SA and SBM led to the lowest overall costs. The contributions of this article are an easy implementable simheuristic for MRP parameter optimization and a promising concept to intelligently manage simulation budget.

## 1. Introduction

For manufacturing companies, an enterprise resource planning system (ERP) is the central system to plan and control production-related resources. Compared to the technological development of ERP systems, the used planning algorithms have not changed that much during the last decade. Most commercial ERP systems still use the hierarchical production planning approach of material requirements planning (MRP) to generate production orders [1]. The applicability in different industries and the straightforward and scalable logic—independent from the product complexity—fostered MRP’s importance in industry and also science. The three planning parameters to control MRP are lot size, planned lead time, and safety stock. Many other parameters must be set up and defined to get a production system on which an MRP can be applied, such as a bill of material (BOM), processing time, setup time, planning period, machine availability, and many more. The master production schedule (MPS), which is based on the customer demands, defines the quantities and time periods of the ordered materials and specifies the gross requirements for the MRP algorithm. Even though the MRP logic is not complicated, keeping an MRP system up to date is a challenging task. Suppose raw material is available in the ERP system, but cannot be handed to production. This type of misinformation can require updating the complete MRP system with re-planning, including scheduled receipts and planned order releases [1]. However, these are not sophisticated tasks compared to selecting optimal MRP planning parameters. To overcome a worsening service level, it is, for example, possible to increase planned lead time with the consequence of increasing stocking level [2]. Considering the effects of changing the value of one MRP planning parameter is manageable, but doing this in a systematic way for lot size, planned lead time, and safety stock need to be supported by methods from the field of production system simulation and heuristics. Using production system simulation to evaluate the performance of a production system relying on an MRP provides interesting insights into the behavior of the production system performance. In particular, the systematic exploration of suitable MRP parameter combinations will help find optimal values with respect to given performance indicators, such as inventory and tardiness costs. Production systems are characterized by a high uncertainty level, which is associated with their planning. This may be due to internal factors, such as stochastic processing times, machine failures, limited availability of resources—either workers or raw materials—etc. It can also be due to external factors associated with customers’ demand, such as required lead times, changes in orders requested, or cancellations [3]. Given this complexity, the planning of the MRP parameters in a production system requires the development of methodologies capable of dealing with systems under uncertainty. The implementation of optimization techniques based on hybrid methods between metaheuristic and simulation techniques has proven to be a methodology with the potential to establish the stochastic MRP parameters at near optimal levels [4]. Therefore, this paper proposes a simulation heuristic (simheuristic) to establish the values of the MRP parameters in a multi-stage, multi-item production system. A discrete event simulation model simulating a stochastic MRP system is combined with a metaheuristic using a daemon-like procedural criterion and simulation budget management (SBM). The stochastic MRP mimics a production plant exposed to the uncertainties associated with stochastic order amounts, the customer-required lead times, and machine setup times. Hereby, the focus of this article is to investigate how to apply different simheuristic approaches to systematically find the best MRP parameter settings in order to minimize overall costs. From an algorithmic perspective, the challenge is to integrate the simheuristics into the used simulation framework. Data from the actual iteration must be stored and provide the foundation for subsequent iterations. For SBM, it is necessary to keep track of previous solution quality. This information is used to decide if the remaining replications of an iteration are consumed. Production system modeling associated challenges are the selection of appropriate starting parameters including the ranges of the MRP parameters. The tackled problem is to identify the combination of MRP parameters providing the lowest overall costs by applying the developed simheuristics. Hence, the performed simulation study is used to answer the following three research questions. To answer these questions the developed simheuristics are evaluated using an MRP-based production system:

Q1: Does the application of an intelligent simulation budget management (SBM) provide lower overall costs compared to a fixed number of replications?

Q2: In the context of MRP parameters setting, can the use of simulated annealing (SA) help avoid getting trapped in a local minimum related to the overall costs?

Q3: Is the combination of SBM and SA leading to lower minimum overall costs and, if so, what is the effect of increasing simulation budget?

The remainder of the article is structured as follows: Section 2 presents a literature review on the use of simulation and simulation-based optimization methods to deal with stochastic MRP systems. Section 3 introduces the simulation-based optimization heuristic used to solve the problem. Section 4 explains the characteristics of the stochastic MRP system used in this work to test our methodology. Section 5 describes the simulation-optimization experiments performed. Section 6 discusses the results obtained. Finally, Section 7 concludes on the findings of this work and discusses future lines of research.

## 2. Related Work

Production systems are characterized by a high complexity given by the large number of parameters involved in their working processes. In general, MRP systems calculate the lot size to be produced or ordered for each component in each period based on the items’ demand. There is a BOM in which they are organized hierarchically as components, and it also includes the number of each part per final product [3]. This section presents a review of some of the works carried out about the optimization of the parameters studied in this work, which are associated with MRP. The objective is to identify the most studied parameters, their combination, and the analysis or solution methods. Please note, that the objective function of the optimization problem discussed in this paper includes inventory and backorder costs. This includes service and time performance effects, i.e., low service level leads to high backorder costs and high production lead times lead to high inventory costs (and probably to higher backorder costs). Consequently, the objective function in this article is in line with a lot of MRP parameter optimization problems from the presented literature.

The MRP in a production line is modeled to simplify the system with a set of assumptions. They include all those parameters that allow us to model the uncertainty and complexity of the system itself. The optimization of these models—either with exact, heuristic, metaheuristic, or even hybrid methods—enables us to find near-optimal and efficient solutions for the system. Among the most studied parameters under uncertainty in the MRP literature are the safety stock and the planned lead time [5]. The approach employed to manage an MRP under uncertainty depends on the type of uncertainty, the significance of its effect, and the managers’ preferences [6]. Table 1 presents some of the optimization works on one or more of the MRP parameters studied in this paper, with their respective solution techniques. From the literature review, it can be concluded that this article tackles the MRP parameter optimization problem, which is already treated by other authors.

Among the related work analyzed, it is found that simulation techniques have been widely applied in MRP models since they allow us to observe the behavior of the supply chain through the creation of experiments based on the model of the system under study. At the same time, it allows us to observe the effects of changes in MRP parameters. It offers the possibility of planning and controlling the production systems and improving strategies at different organizational levels. Likewise, discrete-event simulation mimics the dynamics of systems in the real world, which has made it a very popular method for MRP modeling [17]. Whybark and Williams [7] have been among the first to propose a framework to characterize and study the uncertainty that can affect inventory investment and service level performance in an MRP system. They used simulation to compare two parameters, safety stock and safety lead time. They also tested whether there is better inventory control that meets market uncertainty. In the same direction, Buzacott and Shanthikumar [8], Enns [9] studied the influence of safety stock size and planned lead time. Likewise, these authors developed models that simultaneously decide on the lot size and planned lead time in an environment with constant customer demand and stochastic lead times [18]. Other models are developed to address the risk generated by uncertainty in lead times and demand, while focusing on deciding which of these two parameters should be used to make decisions [10]. The design of these simulation models to explore the effect of demand uncertainty on the system performance also varies depending on whether we are considering a single-stage manufacturing system [2] or a multi-item, multi-stage MRP production system [11].

Typically, MRP simulation models are studied to know the responses (or outputs of the system) to the initially defined parameters [19,20]. However, this technique does not allow determining the optimal values of the parameters to achieve a given objective [21]. Parameter optimization to configure the systems and operate as efficiently as possible is recommended [10]. In the literature, some models establish planned lead times based on capacity requirements planning, using nonlinear optimization to find the optimal values of the planning parameters minimizing production-related costs [12]. Other models rely on simulation-based optimization to perform numerical explorations. The integration of optimization methods and simulation allows us to calibrate the parameters of the stochastic model. In this optimization process, the objective function has an associated measure of the experimental simulation that is optimized [22]. For instance, it includes the optimization of planned stock levels and lead time in production, as well as the optimization of inventory systems with advanced demand information and some service level requirements [13]. There are also analytical models for the simultaneous optimization of capacity and planned delivery time in a two-stage production system with different customer due dates [14]. Another work investigating the application of analytical methods and heuristics was presented in [15]. The inventory and the tardiness costs were analyzed. In addition, a heuristic-based approach to solve the associated optimization problem was used. All three MRP parameters with the limitation of only one production stage and capacity, were investigated.

One of the methods of simulation-based optimization is simheuristics. It is a solution method based on algorithms combining simulation methods within a heuristic/metaheuristic optimization framework. It allows to deal with stochastic combinatorial optimization problems [23,24]. Barrios et al. [4] demonstrated that simheuristics are a promising approach to solve a two-stage stochastic MRP. They identified the safety stock level of each item to minimize the total expected manufacturing cost. Their results demonstrate that the use of simheuristics provides advantages over the use of simulation approaches alone. Some approaches vary between them because of the proposed optimization heuristics and metaheuristics. For example, Molinder [10] proposed to determine the safety lead time with a hybrid approach combining simulation with optimization based on the simulated annealing algorithm. Gansterer et al. [5] presented a simulation-based optimization approach with a search procedure based on variable neighborhood search with a robust production planning as a result. They focused on the simultaneous optimization of three parameters, planned lead time, safety stock, and lot sizes. In addition, Karder et al. [16] optimized the MRP parameters, applied, and compared two different versions of efficient global optimization of single-objective and multi-objective functions. Their results demonstrate how both approaches are competitive with each other.

Thus, some of the works related to the optimization of the MRP system have focused mainly on parameters such as lead time, followed by safety stock and lot size. The literature that has analyzed the three parameters has always used hybrid simulation-based optimization approaches to analyze and solve the model. It is because increasing the number of parameters increases the complexity. The simplest analysis methods usually involve only one or at most two parameters. Simulation is the most widely used analysis method, followed by hybrid simulation-based optimization methods. Moreover, the latter is also one of the most recent solution methods in the literature. Considering the trend of related works, this paper presents a simulation-based optimization methodology to find the values of the studied parameters for MRP that guarantee an efficient performance of the production system, with the optimization target of minimum overall costs.

## 3. Simulation Heuristic

In the context of MRP parameter setting, simheuristic algorithms [25] are a meaningful approach to evaluate a broad range of parameter combinations with a defined objective (e.g., minimizing overall cost). A first simple simheuristic with two extensions to minimize overall costs was presented in [26]. However, their approach is limited in the sense that it excludes lot sizing. Their simheuristic is significantly extended in this paper and applied also for the lot sizing decision which has a major impact on the overall cost. In addition, the new simheuristic algorithm is further integrated into a discrete-event simulation model, which implements a production system relying on MRP. During a simulation experiment, the simheuristic is applied to compute new planning parameters for each planned material. The next simulation iteration is then performed with the changed planning parameters. In Section 3.1, three versions of the proposed simheuristic are described. They all rely on Algorithm 1.

### 3.1. Initial Simheuristic Algorithm

The initial simheuristic depicted in Algorithm 1 allows us to develop different variants, named: static range (STR), exponential range reduction (ERR), and best solution-set base range (BSBR). The initial simheuristic provides a description of the algorithmic flow, and this generic structure facilitates to subsequently integrate the aforementioned variants. The algorithm starts by selecting the best solution of all previously finished iterations. The best solution represents the minimum overall cost of all finished iterations and provides a list with the used mode, lower bound (LB), and upper bound (UB).

For the STR version, a triangular distribution is used to compute new values using the LB, the UB, and the mode given by the midpoint of the range. For all three variants, the possible values for the MRP parameters (planned lead time and safety stock) lie within the LB and the UB. While in [26] the lot size is not included, in this paper, we consider its parameter sampling for optimization in the same way as for the parameters planned lead time and safety stock.

The second simheuristic version, named ERR, updates LB and UB using the best solution. This makes it possible to reach a search space outside the original bounds. The solution range is defined as $r_{0}=$ mode − UB for both MRP parameters. The new bounds for the MRP parameters and for each item are computed as $r_{i}=\left(1-\alpha \right)*r_{i-1}$, where, *i* is the simulation iteration number and α is the range reduction level. The goal of the ERR variant is to reduce the LB and UB when the number of iterations increase, so the possible solution range is iteratively shrinking.

In the third version, named BSBR, the LB and the UB are computed using the minimum and maximum of the best *n* solutions.
**Algorithm 1** Base Simheuristic Algorithm.1:**if**Initialization**then**2:    α←e.g.,0.0253:    topN←e.g.,74:    initIterations←e.g.505:    initParameterRange(lb,ub,mode)6:    n←07:    endInitPhase←false8:    currentSolution←TriangularDistribution(parameterRange)9:    baseSolution←currentSolution10:**end if**11:**while**n≤maxReplications**do**12:    $cost\left(currentSolution\right)\leftarrow Simulate_{MRP}\left(currentSolution\right)$13:    **if** n>initIterations **then**14:        endInitPhase←true15:    **end if**16:    **if** cost(baseSolution)<cost(currentSolution) **then**17:        baseSolution←currentSolution18:    **end if**19:    baseSolution←GetBestSolution()20:    **if** simheuristic=STR∨endInitPhase=false **then**21:        parameterRange.mode←baseSolution22:    **end if**23:    **if** simheuristic=ERR∧endInitPhase=true **then**24:        oldRange←(parameterRange.ub−parameterRange.lb)/225:        newRange←(1−α)∗oldRange26:        parameterRange.mode←baseSolution27:        parameterRange.lb←parameterRange.mode−newRange28:        parameterRange.ub←parameterRange.mode+newRange29:    **end if**30:    **if** simheuristic=BSBR∧endInitPhase=true **then**31:        bestNSolutions←GetBestNSolutions(topN)32:        parameterRange.mode←baseSolution33:        parameterRange.lb←min(bestNSolutions)34:        parameterRange.ub←max(bestNSolutions)35:    **end if**36:    baseSolution←TriangularDistribution(parameterRange)37:    n←n+replicationsPerIteration38:**end while**

The constant α is set for the ERR range reduction and *topN* is required only for the BSBR version. An initialization phase can be applied for both the ERR and BSBR versions. The number of iterations is represented by the constant *initIterations*. The variable *endInitPhase* is used to control the end of the initialization phase. Notice that, by design, ERR and BSBR are extensions of STR. Therefore, STR is applied during the *initIterations*. Thus, for example, 50 iterations out of a maximum of 300 iterations can be used to find starting values for range reduction and the best *n* solutions. A key component in the algorithmic flow is the array *parameterRange*, as it represents the starting LB and UB values, as well as the mode for each MRP parameter and planned item. In addition, this array is continuously updated during the simulation. The final step of each iteration in each version is to pass the adapted *parameterRange* to the triangular distribution. This allows us to generate new MRP parameter values and apply them during the MRP simulation. At the beginning of each iteration, the base solution is updated to the *currentSolution* in case this is the new best-found solution. The best solution is represented by the minimum overall cost of the previous iterations, computed using the average overall cost of the simulation runs. The objective is to minimize overall costs. A limitation is that logistic objectives such as service level and lead time are not considered. Even though the overall cost criteria are common in a lot of studies (see Section 2, e.g., [5,13,15]), this limitation implies that the effect of the optimized planning parameters on other important key performance indicators is not analyzed which restricts the discussion to one dimension.

The algorithm is repeated until a maximum number of runs (*maxReplications*) is reached. Hence, for instance, for 100 iterations with 20 runs, the value for *maxReplications* is 2000. The statement $Simulate_{MRP}$ is then performed 20 times per iteration.

As shown in [26], even basic versions of these concepts can be useful to identify parameter settings that minimize the overall cost. In this paper, we extend the initial concepts to develop a holistic approach for MRP parameter optimization. Thus, the following novel aspects have been considered in our study: (i) the MRP parameter lot size has been added; (ii) the original heuristic has been extended into a full simheuristic; and (iii) an intelligent simulation budget management is included. The target of the simulation budget management is to test solution quality after each run within an iteration. In other words, for each parameter set, the overall cost is compared to the past results, and only if the solution is sufficiently good, further runs of the current iteration are conducted. The target is to perform more iterations with the same overall number of runs—i.e., the same simulation budget—by avoiding unnecessary runs.

### 3.2. Simulated Annealing

Escaping a local minimum and exploring a new solution range requires a concrete strategy. A biased-randomization algorithm [27] of triangular distribution mode is described in Algorithm 2. This algorithm applies aspects of the SA framework [28]. In addition, the freedom aspect of a demon algorithm is used. Such a demon-based behavior is explained in [29]. After each iteration, the costs of the base solution are compared to the costs of the best solution. Each time the costs of the base solution are greater or equal to the best solution, a reset counter is decreased by one. When the reset counter reaches 0, the base solution is reset to the best solution. The reset to the best solution avoids wasting simulation budget in a not promising solution space.
**Algorithm 2** Reactive biased-randomization of triangular distribution mode.1:**if**Initialization**then**2:    resetCounter←initialValuee.g.,53:    bestSolution←baseSolution4:**end if**5:δ=cost(baseSolution)−cost(currentSolution)6:**if**δ>0**then**7:    credit←δ8:    baseSolution←currentSolution9:    **if** cost(baseSolution)<cost(bestSolution) **then**10:        bestSolution←baseSolution11:    **end if**12:**else if**−δ≤credit**then**13:    baseSolution←currentSolution14:    credit←015:**end if**16:**if**cost(currentSolution)≥cost(bestSolution)**then**17:    resetCounter−−18:**end if**19:**if**resetCounter=0**then**20:    baseSolution←bestSolution21:    resetCounter←initalValue22:**end if**

### 3.3. Simulation Budget Management (Sbm)

Limiting the simulation experiment by a maximum number of iterations and runs per iteration increases the probability to exclude potential good solutions, as they are excluded due to the run out of simulation iterations. Always performing a constant number of runs per iteration will not necessarily increase the potential to find a new best solution. Consequently, it would be more meaningful to introduce a constraint for skipping the actual iteration and invest the remaining simulation budget to explore new solutions with additional iterations and a changed set of parameter values. A simple simulation budget management pseudocode, which facilitates to run the simulation experiment, is described in Algorithm 3. This algorithm allows as many iterations as budget is available, and will stop the last iteration when the maximum number of runs is reached. The best solution always has the maximum number of the given replications due to the stopping criteria. The average cost of the previous iterations (*avgIterationOverallCosts*) is equal to $\frac{1}{n}\sum_{i}^{n}overallCosts_{i}$, with n= maximum runs per iteration (*maxReplicationsPerIteration*). The *avgIterationOverallCosts* is compared to the percentile value of all past iterations, which depends on the replication number. The percentile value is computed using *GetPercentilValue(setOfAllSolutions, β)*, which requires the previous overall cost to be equal to *setOfAllSolutions*, and a value reduced after each run from a UB, returning the value associated with the position in the passed solutions. These values are represented by β and *percentileStep*. After each replication, the overall cost is added to the values of the current iteration and the new percentile value is computed. Compared to the initial Algorithm 1, we do not always perform a fixed number of runs. The SBM stops after the *simulationBudget* is exhausted.
**Algorithm 3** Simulation budget management.1:**if**Initialization**then**2:    totalReplicationCount←03:    simulationBudget←maxIterations∗maxReplicationsPerIteration4:    stopCurrentIteration←false5:    currentReplicationCount←16:    percentileStep←0.01757:**end if**8:β←0.49:**while**currentReplicationCount≤maxReplicationsPerIteration ∧10:    stopCurrentIteration=false **do**11:    totalReplicationCount++12:    currentReplicationCount++13:    **if** avgIterationOverallCosts>GetPercentilValue(setOfAllSolutions,β)∧currentReplicationCount>3 **then**14:        stopCurrentIteration←true15:    **end if**16:    β=β−percentilStep17:**end while**18:stopCurrentIteration←false19:replicationsPerIteration←currentReplicationCount

## 4. Modeling a Stochastic Mrp-Based Production System

To model a stochastic MRP system, discrete event simulation can be used [30]. From a technical perspective, this requires agents to simulate the customers’ orders behavior and a simulation framework capable of reading the given experiment planning parameters and change them after each iteration. The experiment planning parameters include start values and boundaries for the MRP-related planning parameters of lot size, planned lead time, and safety stock. In addition, MRP related settings, such as planning horizon and additionally technical parameters are required for the simulation experiments, such as the number of simulation runs and algorithm iterations [31]. Customer’s order agents are passed to a queue. Whenever a customer’s order cannot be completed before the deadline, it is classified as ‘delayed’. The same logic is applied for the production orders. The production orders are generated based on customer’s orders, which are the gross requirements for the applied MRP run. The output of each MRP run is the production orders, which provide the information on quantity, item, start and end date. After each iteration the solution quality is evaluated, i.e., the average of all replications’ overall costs is calculated, and the simheuristic is applied to set the new MRP planning parameter values for the next iteration. The simulation results of each iteration (MRP parameters, backorder costs, inventory costs, iteration count, replication count, …) are stored after each iteration in the linked in-memory database. These values are then available for the subsequent iterations. The results of the current iteration including each replication are hold in the working memory of the simulation computer, until the last replication is performed. The results of the in-memory database are stored as database file and are the basis for the performance analysis of the undertaken simulation study.

### 4.1. Stochastic Mrp Setting

Our MRP simulation model can handle stochastic demands as well as random processing times, and provides the described standard MRP logic to treat customers’ orders. Stochastic behavior is introduced and controlled using log-normal probability distributions with expected values ($\mu_{i}$) and variances ($\sigma_{i}^{2}$) for machine setup time, customer required lead time and customer’s expected order amount. The random variables can be found in Table 2, which also includes the respective coefficients of variation ($CV_{i}$).

**Figure 1:**
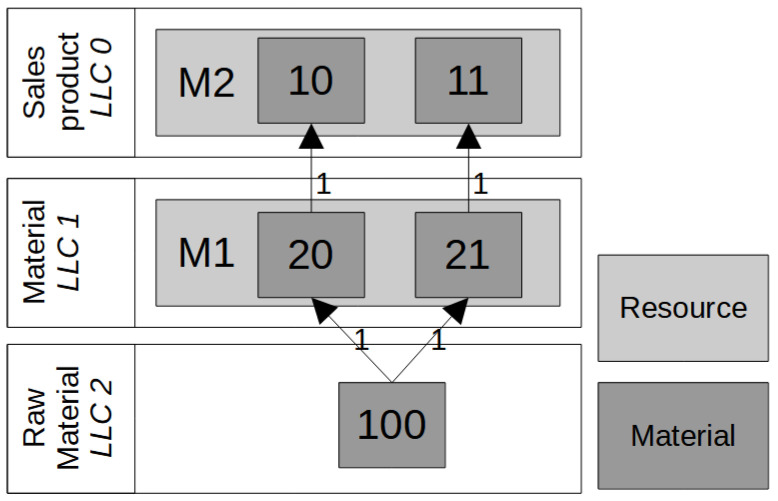
A *3*-level BOM applied during stochastic MRP simulation.

The simulated production system is illustrated in Figure 1. The role of the developed simheuristic is to set lot size, planned lead time and safety stock parameters in order to minimize the overall cost, which is the sum of inventory and tardiness costs. Despite being a simple BOM that considers only three levels, the example allows to demonstrate how simheuristics can be meaningfully integrated into an MRP system to optimize overall costs for all three planning parameters. Two final products, products 10 and 11, at low-level-code (LLC) 0 are produced on machine M2. The two semi-finished products, materials 20 and 21, are produced on machine M1. For one unit of final product 10, 1 semi-finished product 20 is needed. One piece of semi-finished product 21 is needed for final product 11. The raw material 100, which is a purchased product, is needed for the semi-finished products 20 and 21. This raw material is assumed to be always available. For this simple production system, 12 different parameters have to be optimized because during simulation for each of the materials (10, 11, 20 and 21), all three MRP planning parameters have to be tested for optimality. Table 3 shows the start-up values and respective ranges for optimization parameters. Please note that the MRP planning parameter values are treated and evaluated as integer units. This means that in the simulation results, the increase or decrease of overall costs related to each individual MRP parameter can be tracked. Furthermore, the interrelation between the single planning parameters is integrated in the simulation model, e.g., both a higher planned lead time and a higher safety stock lead to higher inventory costs and lower tardiness costs, also compare to Buzacott and Shanthikumar [8]. A decrease in lot size, for example, leads to higher setup efforts and, therefore, also affects the production lead time. A higher safety stock increases inventory costs and also increases service level which leads to lower backorder costs.

### 4.2. Mrp Procedure

During the execution of the MRP logic, the netting, lot-sizing, backward scheduling, and BOM explosion steps are subsequently performed for the specified planning horizon. For the netting step, the outputs are the items to produce, which are not yet organized as production lots. For the subsequent lot sizing step, the required information is a lot sizing policy, i.e., the fixed order quantity (FOQ) and the associated parameter value representing the amounts for the production lot. One of the targets is to reduce setup times by selecting efficient lot sizes. The process of machine setup plays an important role in the context of lot sizing. The planned lead time is the required input for the step of backward scheduling. With backward scheduling, the start date of a production order is computed. The outputs of the last step (BOM explosion) are the required quantities for the subsequent BOM levels. The items of BOM level 0 are processed, and followed by the process of higher BOM levels. Notice that level 0 provides the gross requirements for subsequent levels: the required quantities for BOM level *n* are passed to BOM level n+1. Our actual research activities are focused around MRP and it is, therefore, the selected production planning approach for this publication. MRP is still widely applied in industry and the target of current research activities. Therefore, this article will contribute to MRP-related research activities and provides also input for practitioners of systematically setting MRP planning parameters with respect to minimizing inventory and backorder costs.

### 4.3. Simulation Model with Simheuristics

The proposed simheuristic versions are integrated into the simulation model of a stochastic MRP-based production system. The computation of all three MRP parameters is considered, and SA is used to set a new base solution. In addition, SBM can be activated for the simulation process to consume the available simulation budget only for promising solutions. One of the goals of the numerical experiments will be to test the performance of the SA and SBM procedures as an extension of the STR, ERR and BSBR. A simulation experiment starts at time 0 and lasts until $t_{n}$. In a rolling-horizon manner, the customer’s orders are updated and integrated into the MRP procedure. The planning frequency and planning horizon must be set for MRP. The planning frequency determines how often the MRP execution is applied, while the planning horizon determines how far in the future the MRP planning is performed. The frequency was set to 1 day, and the horizon to 70 days. A complete MRP re-planning is possible and suitable in the developed simulation framework. From $t_{i}$ until $t_{i+70}$, the gross requirements are used to compute production orders. If an initialization phase is set, the selected simheuristic version is applied after the initialization iterations are consumed and until *maxReplications* is reached. For the simulation experiment, a set of parameter values is required to provide the required information during the initialization of the simulation experiment. As illustrated in Table 3, the parameterization of the simheuristic versions (including LB, UB and mode for the used triangular distribution) must be defined for all three MRP planning parameters. The used lot sizing policy is FOQ. The rows with LB, UB and mode represent the setup values which are passed to the simulation model at the starting time, which are therefore used in the initialization phase. The mode is changed for all three versions, while the LB and the UB only for ERR and BSBR.

## 5. Simulation Study

To evaluate the performance of the proposed approach, a computational study was performed. During the simulation experiments, the simheuristic versions are applied to find and update the base and best solutions and set them for the remaining iterations. The simulation results are stored in an in-memory database, and are afterwards used to analyze the simulation results. To reduce the variance in the simulation results, outliers (runs with very high or very low costs of an iteration), were excluded from the average overall cost per iteration. The simulation time was set to 1800 days and 20 replications per iteration are applied when SBM is not activated. Different amounts of simulation runs were evaluated to compare the performance of the different simheuristics. Based on the existing simheuristic versions, the developed SBM and SA extensions were evaluated during the simulation study with 100, 200 and 300 iterations, i.e., a *simulationBudget* of 2000, 4000 and 6000 runs. To get promising settings for the the remaining parameters, a set of preliminary test simulations was performed. For the STR, no additional simulation parameter is required. For the ERR, the α value for the range reduction was set to 0.0025, thus avoiding a too quick convergence of the LB and UB. For the BSBR, the best 7 solutions are used to compute lower and upper parameter bounds.

To illustrate the simhueristic application, one optimization run is described here in detail. The starting values for the MRP parameters are: item 10 [FOQ 600, SS 3, PL 2], item 11 [FOQ 700, SS 3, PL 2], item 20 [FOQ 450, SS 3, PL 2] and item 21 [FOQ 450, SS 3, PL 2]. These MRP parameter values are applied for the first iteration within the optimization run and lead to the initial overall costs. Note that all MRP parameter values generated by the simheuristic are firstly calculated as double values and then rounded to be applicable for the simulation model. In the presented example, the STR simheuristic is applied with 100 iterations. The MRP parameters lead to initial overall costs of 7151 CU with inventory costs of 1697 CU and backorder costs of 5454 CU. This is also our base and best solution used for the subsequent iteration. Applying the STR heuristic for the second iteration leads to the following MRP parameter values: item 10 [FOQ 778, SS 4, PL 2], item 11 [FOQ 678, SS 2, PL 3], item 20 [FOQ 452, SS 5, PL 1] and item 21 [FOQ 470, SS 3, PL 2]. These values result in deceasing overall costs of 6343 CU (1888 CU inventory costs, 4455 CU backorder costs).

This process of finding new base and best solutions with the associated MRP parameter values is done until the maximum number of iterations, i.e., 100 in this example, is reached. When SBM is applied, the number of iterations is limited by the overall number of simulation runs. Finally, the minimum overall costs can be computed and used for comparison with other simulation results. In this example, the minimum overall costs are 3490 (1899 CU inventory costs, 1591 CU tardiness costs) at iteration 55 with parameter values of item 10 [FOQ 539, SS 5, PL 2], item 11 [FOQ 615, SS 5, PL 3], item 20 [FOQ 478, SS 3, PL 3], item 21 [FOQ 427, SS 4, PL 2]. Compared to the initial solution, this is a decrease of the overall costs by 3661 CU, the inventory costs increased by 202 CU and the backorder costs decreased by 3863 CU. Comparison of MRP parameters of the initial solution and the best solution shows a decrease in the lot sizes, an increase in the safety stock for items 10, 11 and 21 and an increase in the planned lead time for item 11 and 20. Please note that the safety stock parameter is passed as factor to the MRP run and multiplied by associated stock units.

## 6. Results of the Simulation Study

In order to investigate the performance of the proposed simheuristic versions, a simulation study was performed. The target was to identify the impact of SBM and SA on the minimum overall cost that can be achieved. Each simheuristic version (STR, ERR, and BSBR) was evaluated using 100, 200 and 300 iterations, while each combination of SBM and SA was tested. The 36 possible experiment combinations and their associated minimum overall costs are shown in Table 4. Each result represents a unique and not completely reproducible simulation experiment. Each experiment was performed four times to get comparable results and to lower the impact of outliers. For example, from Table 4, the experiment E= {STR with SBM = NO, SA = YES, Iterations = 100} was run four times to get the value of 3159 cost units (CU). This value represents the average minimum overall cost of this four experiments. The result of the previous experiment used the simulation budget of 2000 runs. In detail, for 100 iterations the simulation budget is 2000 runs, for 200 iterations the budget is 4000 runs, etc. In total, 768,000 runs were performed to get the results described below.

**Figure 2:**
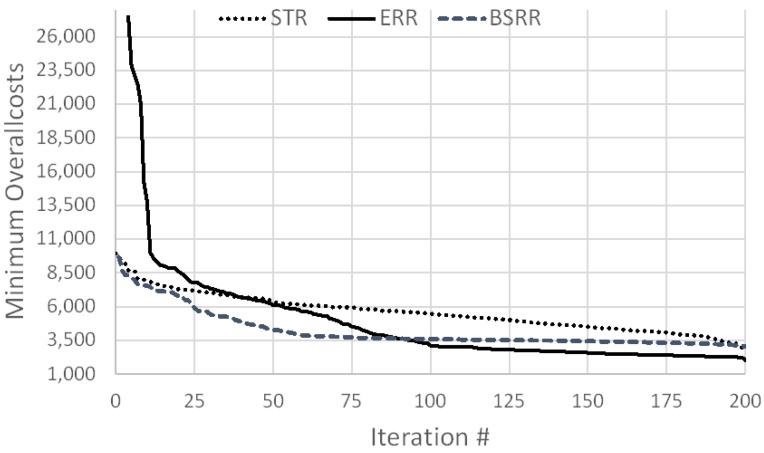
Minimum overall cost per simheuristic version for all MRP planning parameters.

To analyze the effects of setting values for lot size, the STR, ERR and BSBR versions are evaluated using a simulation experiment with 200 iteration, 20 runs and simulation experiment run time of 1800 days. The results of the associated simulation experiments are illustrated in Figure 2 and represent the applied basic heuristics setting from [26] with all three MRP parameters optimized. In the scenario comparison, Table 4, the results correspond to experiment F= {SBM = NO, SA = YES, Iterations = 100}. Notice that the ERR line was cut to get a comparable diagram with STR and BSBR. Consequently, the minimum overall costs are higher than 26,000 CU in the first iterations. Even though ERR has clearly a higher minimum overall cost at the beginning of the optimization experiment, after 200 iterations, it leads to the lowest overall costs. Looking at STR, the minimum overall cost gradually decreases over all 200 iterations. Similarly, considering BSBR from iteration 100 to 200, the minimum overall cost changes only slightly. These results fit to the expected behavior of the three simheuristic versions. The STR always uses the complete solution space between the lower and upper bounds of the MRP parameters for the whole simulation experiment run time. In contrast, for ERR, the LB and UB are updated and scaled down each time a new best solution was found. For BSBR, the decreasing performance can be argued due to the fixed set of top *n* solutions defining LB and UB for the MRP parameters. If the setting of the top *n* solutions is not changed, the same LB and UB are used.

### 6.1. Sbm Application

Our first research question (Q1) investigates the performance when applying SBM. The idea of SBM is to stop an iteration if the computed average costs of the current iteration’s run is higher than a specified threshold based in the prior available results. The wasting of simulation budget, represented by additional non-promising runs, should be avoided. The leftover runs are shifted to the next iteration and, consequently, allow more iterations per optimization experiments with different parameter settings. To answer Q1, the results without SBM {SBM = NO and SA = NO} and applying SBM {SBM = YES and SA = NO} in Table 4 are compared. For the STR procedure, the application of SBM and an increasing simulation budget indicates a lowering overall cost. At 100 iterations, the overall cost decreased from 3482 CU to 3100 CU, which is a performance increase of 10.95%. Likewise, at 200 and 300 iterations, a cost reduction of 11.31% and 13.89% can be observed, respectively. For ERR, at 100 iterations a better performance of 6.18% is possible. At 300 iterations, a performance increase of 8.21% (from 2173 CU down to 2118 CU) is observed. For simheuristic BSBR, the application of SBM with SA = NO does not show a consistent behavior, although costs for 300 iterations slightly decrease. Note that the BSBR version is outperformed by the ERR one in all of the aforementioned scenarios. For Q1, it can be concluded that the SBM application leads to a performance increase, when the search space is focused on a single best solution and a constantly decreasing parameter space leads to an additional performance increase.

### 6.2. Sa Application

The second research question (Q2) addresses the problem of getting stuck in a local minimum. The application of SA should help escape a local minimum by exploring other regions of the solution space. The pseudocode described in Algorithm 2 facilitates this by computing the delta between current and base solution. Only when delta is positive, a new best solution can be found. To test the performance of applying only SA, the results without SBM and SA {SBM = NO and SA = NO} are compared to results with SA {SBM = NO and SA = YES}, which can be found in Table 4. For the simheuristic STR with 100 iterations, an improvement of 9.26% (3482 CU vs. 3159 CU) is obtained. In addition, with 300 iterations, an improvement of 7.90% (3286 CU vs. 3026 CU) could be obtained. For ERR with 200 iterations, the overall cost decreases by 4.59%, while for 300 iterations, it decreases by 2.36%. For each maximum iteration set in BSBR, the overall cost decreases step-wise by 0.53% (3201 CU vs. 3184 CU) for 100 iterations, by 5.12% (3066 CU vs. 2909 CU) for 200 iterations, and by a remarkable 13.17% (3257 CU vs. 2828 CU) for 300 iterations. Hence, for Q2, the conclusion is that the application of SA leads to decreasing overall costs with an increasing simulation budget. In addition, the combination with SBM may support this trend. The results in Table 5 support this finding, with the lowest average of 2693 CU for a simulation budget of 300. The columns STR, ERR and BSBR represent the average minimum overall cost over the same simulation budget for each simheuristic, while the last column shows the average per simulation budget.

### 6.3. Combination of Sbm and Sa

The third research question (Q3) investigates the joint application of SBM and SA to compute the minimum overall cost. The joint application of SBM and SA led to the lowest overall costs of all experiments, with a value of 1947 CU and a performance increase of 15.64% for ERR (from a value of 2308 CU, without SBM and SA), as shown in Table 4. The highest decrease of 22.47% of the overall cost (compared to the experiments without applying SA and SBM) could be obtained with 300 iterations and BSBR (from 3257 CU down to 2525 CU). For STR with 300 iterations, it was possible to lower the overall cost by 17.22% (from 3286 CU to 2720 CU). For Q3, the main conclusion is that an increasing simulation budget, and the joint application of SBM and SA, provides an obvious performance increase compared to the experiment where only SBM or SA is applied, and even higher if both are excluded. In addition, the overall results show that the most competitive simheuristic version for this application is ERR, which significantly outperforms the other versions.

### 6.4. Discussion of Best Simulation Result

As described in Section 5, each simheuristic starts with the same initial parameter values. The lowest overall costs from Table 4 are 1947 CU (ERR, SBM YES, SA YES, 300 iterations) which are based on three different replications of the respective simheuristic. This means that the values in Table 4 show the average of 3 optimization runs applying the respective simheuristic; therefore, the discussion here is based on the lowest overall costs reached by the best optimization run. These best overall costs are 1872 CU (inventory 1746 CU, tardiness 126 CU) with the following MRP parameters: item 10 [FOQ 477, SS 5, PL 3], item 11 [FOQ 500, SS 5, PL 3], item 20 [FOQ 477, SS 5, PL 2] and item 21 [FOQ 55, SS 4, PL 3]. Comparing the initial solution to the best solution shows a high decrease in the tardiness costs. The lower tardiness costs are expressed by smaller lot sizes of the finished goods and sub-item 21, increasing safety stock for all four items and an increasing planned lead time with the exception of item 20. This value constellation is the best to absorb the existing production system uncertainty and keep a high service level. The very low lot size of 55 from the best solution is interesting, as this means more production lots must be produced and this increases setup costs. Since all cost and MRP parameter values of the simulation experiments are available, an interesting analysis for further research would be on the consistency of the MRP planning parameters for a set of best results, e.g., analyzing the 10 best iterations. This leads to additional managerial insights from a production planning point of view. In further research studies, it would also be possible to consider setup costs as additional optimization parameter, which can lead to other parameter constellations.

### 6.5. Statistical Interpretation of Simulation Results

**Figure 3:**
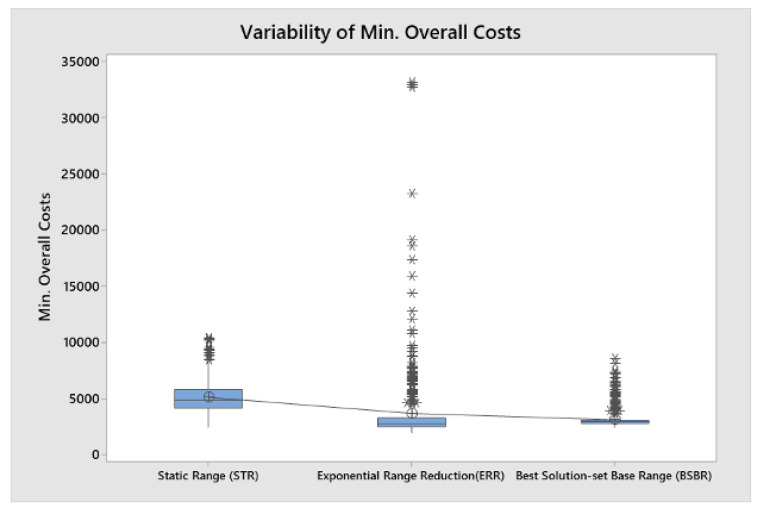
Comparison between the best result obtained in each simheuristic.

Figure 3 presents a set of box-plots that allow us to visualize and compare the performance of each of the simheuristic versions with respect to the minimum overall cost per iteration obtained by each one, e.g.,: STR with 2720 CU, ERR with 1947 CU and BSBR with 2525 CU. This result allows us to reinforce different conclusions already presented. Given the stochastic nature of the variables of the problem, there is a risk that the minimum iteration cost will rise above the expected values when applying any of the versions, as confirmed by the outliers observed for each of them. The results of ERR show the lowest overall median, but with the known risk of the higher variability of the results. On the other hand, the STR version presents a higher mean and median, but at the same time, there is more control over the results due to the symmetry present in the distribution of the results, and there is a lower probability of obtaining outliers compared to the others. BSBR offers higher risk solutions with respect to outliers. However, the interquartile range is very small, and 50% of the data is less dispersed, which represents more control over the processes. Furthermore, comparing the results of ERR with BSBR, it could be said that the outliers in BSBR are mild. From the point of solution quality, the higher variability of ERR allows for identifying lower overall costs.

### 6.6. Implications on Practitioners and Further Research

Researching on MRP planning parameter settings affects researchers and practitioners. From a practical point of view, it would be interesting to implement the previously described pseudocodes into the production planning routine of an ERP or MES (Manufacturing Execution System) system, when such a system also provides a simulation framework for the respective production system. For ERP/MES integration, a main challenge will be the application on an existing and more complex product structure with much more MRP planning parameters, i.e., more decision variables. This requires to upscale the system for parameter handling and intermediate result computation. Furthermore, an efficient MRP parameter update strategy has to be developed. Results of such an implementation with real data can provide valuable results about optimal parameter combinations and the performance issues of the respective production system. The automatic computation of optimal MRP planning parameter based on optimized inventory and backorder costs provides useful information of production planning related decision making. From a scientific point of view, especially the general approach of SBM is reusable for other simulation studies and can help consume the available simulation budget to test larger solution spaces.

The presented simheuristic does not explicitly consider the interrelation between optimized planning parameters. Ignoring the parameter dependency can have negative effects on the optimized parameters and the final result. However, parameter interrelation is also ignored by other heuristics, compare with [16]. Investigating MRP parameter interrelation may provide additional insight into optimization-based production system simulations, but is out of context of this article.

## 7. Conclusions and Future Work

This article provides insights into the application of simheuristics in identifying optimal MRP planning parameter values for lot size, planned lead time and safety stock. The aim is to find the minimal overall cost (inventory costs plus backorder costs) computed during the application of the MRP planning algorithm in a simulation environment. This article is a further development of the results based on three different simheuristic versions to identify the minimal overall cost only for the MRP planning parameter safety stock and planned lead time. The first extension is that optimal values for all three MRP planning parameters are searched. A second extension is the development of a SA-based simheuristic extension. The third extension focuses on finding optimal parameters without wasting simulation budget for non-promising iterations. Hence, an intelligent simulation budget management (SBM) is introduced to consume the available replications only for potential best solutions, thus skipping solutions outside the defined percentile range. SBM was combined with the SA extension to additionally explore new areas of the solution space and to escape local minima. The application of the developed SBM and SA extensions, combined with all three planning parameters, allows for a systematic identification of optimal planning parameters with a clear optimization target. The numerical results show that the best performing version for this application is ERR. From a production planning perspective, the developed simheuristic versions, specifically ERR, provides a fast and well performing simulation-based optimization approach which is simple to apply in practical environments. The performed simulation study shows the improvement of the existing simheuristic versions regarding the identification of the minimal overall cost with the joint application of SBM and SA. This finding is supported by the lowest overall cost obtained with the highest simulation budget and the joint application of SBM and SA for all three simheuristic versions. Limitations of this study are related to the application field and the production system within which the simheuristics are tested, i.e., only one application field is evaluated in this study and the respective production system is still rather simple. However, this article provides the foundation for future investigations, to systematically apply the simheuristics concept to more complex production system structures and to other application fields. The developed simheuristics focus on a single objective function, however, for real production systems, it is not always possible to transform all objective dimensions into costs. Therefore, future work could investigate how to extend the simheuristic to a multi-objective approach. A broad sensitivity analysis to prove the promising results of SBM is also planned in future work.

## Figures and Tables

**Table 1:** Related work reviewed.

Author	Approach Parameters				Method of Analysis
	Lead Time	Safety Stock	Lot Size	Demand	
Whybark and Williams [7], Buzacott and Shanthikumar [8], Enns [9]	X	X		X	Simulation
Molinder [10]	X	X	X	X	Hybrid: simulated annealing & simulation
Altendorfer [2]	X		X		Heuristic
Altendorfer et al. [11]			X	X	Simulation
Teo et al. [12]	X			X	Non-linear optimization
Liberopoulos and Koukoumialos [13]	X	X			Simulation
Altendorfer and Minner [14]	X			X	General optimization model
Altendorfer [15]	X	X	X		Heuristic
Barrios et al. [4]		X			Hybrid: heuristic & simulation
Gansterer et al. [5]	X	X	X		Hybrid: Variable neighbor search & simulation
Karder et al. [16]	X	X	X		Hybrid: Genetic Algorithm & simulation

**Table 2:** Random variables with log-normal behavior.

Parameter	Item	$\mu_{i}$	$\sigma_{i}^{2}$	${\mathrm{CV}}_{i}$
order amount	10	10	2	0.1414
order amount	11	15	6	0.1633
customer required lead time	10	6	9	0.5000
customer required lead time	11	6	10	0.5270
machine setup time	all	12	36	0.5000

**Table 3:** Values of the simulation parameters employed.

	Material			
Parameter	10	11	20	21
FOQ: LB; UB; Mode	400; 800; 600	600; 800; 700	400; 500; 450	400; 500; 450
SS: LB; UB; Mode	1; 5; 3	1; 5; 3	1; 5; 3	1; 5; 3
PL: LB; UB; Mode	0; 4; 2	0; 4; 2	0; 4; 2	0; 4; 2
Processing time in h	0.17328	0.17328	0.17328	0.17328
Holding costs per day	1	1	0.5	0.5
Tadiness costs per day	19	19		
Avg. demand per day	47	70	47	47
FOQ = Fixed Order Quantity; LB = Lower Bound; UB = Upper Bound; SS = Safety stock; PL = Planned lead time				

**Table 4:** Scenario comparison using minimum overall costs per simulation experiment.

SBM NO			SBM NO		
**Iterations**	**Simheuristic**	**SA NO**	**Iterations**	**Simheuristic**	**SA YES**
100	STR	3482	100	STR	3159
	ERR	2265		ERR	2409
	BSBR	3201		BSBR	3184
200	STR	3155	200	STR	3228
	ERR	2173		ERR	2073
	BSBR	3066		BSBR	2909
300	STR	3286	300	STR	3026
	ERR	2308		ERR	2253
	BSBR	3257		BSBR	2828
**SBM YES**			**SBM YES**		
**Iterations**	**Simheuristic**	**SA NO**	**Iterations**	**Simheuristic**	**SA YES**
100	STR	3100	100	STR	3022
	ERR	2125		ERR	2081
	BSBR	3458		BSBR	2767
200	STR	2798	200	STR	2970
	ERR	2225		ERR	2180
	BSBR	3284		BSBR	2549
300	STR	2830	300	STR	2720
	ERR	2118		ERR	1947
	BSBR	3222		BSBR	2525

**Table 5:** Average performance comparison of simulation budget.

Simulation Budget	STR	ERR	BSBR	Avg
100	3191	2220	3152	2854
200	3038	2163	2952	2717
300	2965	2156	2958	2693

## Data Availability

Not applicable.

## Abbreviations

The following abbreviations are used in this manuscript:

BOM	Bill of Material
CU	Cost Unit
CV	Coefficient of variation
ERP	Enterprise Resource Planning
FOP	Fixed Order Period
FOQ	Fixed Order Quantity
LB	Lower Bound
MRP	Material Requirements Planning
UB	Upper Bound
SA	Simulated Annealing
SBM	Simulation Budget Management
